# Reliable Detection of Excessive Sperm Ros Production in Subfertile Patients: How Many Men with Oxidative Stress?

**DOI:** 10.3390/antiox13091123

**Published:** 2024-09-18

**Authors:** Costanza Calamai, Elena Chelli, Oumaima Ammar, Michele Tanturli, Linda Vignozzi, Monica Muratori

**Affiliations:** 1 Department of Experimental and Clinical Biomedical Sciences “Mario Serio”, University of Florence, Viale Pieraccini, 6, I-50139 Florence, Italy; costanza.calamai@unifi.it (C.C.); elena.chelli1@edu.unifi.it (E.C.); michele.tanturli@unifi.it (M.T.); linda.vignozzi@unifi.it (L.V.); 2 Department of Health Sciences, Section of Obstetrics and Gynecology, Careggi Hospital, University of Florence, I-50134 Florence, Italy; oumaima.ammar@unifi.it; 3 Andrology, Women’s Endocrinology and Gender Incongruence Unit, AOU Careggi, I-50134 Florence, Italy

**Keywords:** oxidative stress, male infertility, routine semen analysis, sperm DNA fragmentation, leukocytospermia, semen viscosity, bacteriospermia

## Abstract

Sperm oxidative stress has been extensively associated to male infertility. However, tests to detect this parameter have not been yet introduced in clinical practice and no definitive data are present on the extent of oxidative stress in male infertility. In this study, we used a novel and reliable flow cytometric method to reveal sperm ROS production in subfertile patients (n = 131) and in healthy donors (n = 31). Oxidative stress was higher in subfertile patients (14.22 [10.21–22.08]%) than in healthy donors (9.75 [8.00–14.90]% (*p* < 0.01)), but no correlation was found with age, semen quality or sDF. We also failed to detect an increase in sperm ROS production with semen viscosity or leukocytospermia, but a sharp impact of semen bacteria was evident (with bacteria: 31.61 [14.08–46.78]% vs. without bacteria: 14.20 [10.12–22.00]%, *p* < 0.01). Finally, after establishing a threshold as the 95th percentile in healthy donors, we found that 29% of subfertile patients exceeded this threshold. The percentage decreased to 25.56% when we excluded subjects with bacteriospermia and increased to 60.87% when only these patients were considered. In conclusion, 29% of subfertile patients showed an excessive sperm ROS production. Surprisingly, this parameter appears to be independent from routine semen analysis and even sDF determination, promising to provide additional information on male infertility.

## 1. Introduction

Male infertility varies from 4.5 to 12%, with the highest values in Europe and Australia [1], and accounts for approximately 30–50% of all infertile couples [2]. A large percentage of infertile men are idiopathic or unexplained, meaning that the causes remain obscure. In idiopathic male infertility, semen quality is impaired but physical examination and hormonal levels are normal and there is not a previous history of reproductive issues. In unexplained infertility, a female factor can be ruled out and men show normal semen parameters, remaining undiagnosed because of lack of clinical findings. According to several authors [3,4,5], a relevant percentage of cases of idiopathic and/or unexplained male infertility would be due to oxidative stress, a condition where the redox balance is perturbed by an excessive presence of oxidant molecules, including reactive oxygen species (ROS). Indeed, as indicated by studies on humans and animals, many environmental [6,7,8,9,10,11], lifestyle [12,13,14,15,16,17,18,19,20,21,22,23,24,25,26] and endogenous factors [27] known to impact male fertility are believed to act by inducing oxidative stress; however, the exact mechanism remains elusive. Spermatozoa are quite vulnerable to ROS because they lose most cytoplasmic antioxidant enzymes during spermiogenesis, show high levels of membrane poly unsaturated fatty acids prone to lipid peroxidation and do not have DNA repair mechanisms [28].

Accordingly, numerous studies have tested treatments with antioxidants for male infertility, reporting beneficial effects in several cases [29,30,31,32,33]. However, the evidence is insufficient to recommend the clinical use of antioxidants [34], which may even cause deleterious effects on sperm functions when administrated in improper doses. Indeed, it is well known that small and time-regulated amounts of ROS play an important role in crucial processes such as the maturation, capacitation, hyperactivation and acrosome reaction of spermatozoa [35,36,37]. In addition, excessive or unnecessary exposure to antioxidants alters redox balance and may lead to reductive stress, a condition also deleterious as oxidative stress [38]. In this scenario, it is clear that it is of upmost importance to assess the individual’s seminal redox state both before starting the treatment and during the follow up of the patient. Hence, as recently discussed [39], there is an urgency to develop validated assays able to reveal sperm oxidative stress.

Our group recently showed a novel flow cytometric method to detect excessive ROS production by the viable sperm fraction of native semen samples [40]. Native semen samples are more representative of the in vivo conditions than selected sperm populations, used by most flow cytometric methods. In addition, the viable fraction is the most important one from a clinical point of view, as well as to represent the real target of oxidative attack [41]. In previous studies, we showed that this method is able to reveal the oxidative burst accompanying the freezing/thawing processes during sperm cryopreservation [42] and the spontaneous increase of sperm ROS generation during short in vitro incubations [40]. Further, with this method, we showed that cancer highly increases sperm oxidative stress with respect to normozoospermic subfertile subjects and healthy donors [43].

The aim of this study was to use the above novel method for measuring excessive sperm ROS production in male partners of infertile couples attending our clinics to undergo routine semen analysis. After establishing a threshold value in healthy donors, we determined how many men exhibited high levels of sperm oxidative stress. We also evaluated the relationship between sperm ROS production and putative signs of semen oxidative stress, standard semen parameters and sperm DNA fragmentation (sDF) amounts.

## 2. Materials and Methods

### 2.1. Reagents and Media

Human Tubal Fluid (HTF) was purchased by Fujifilm, Irvine Scientific (Rome, Italy). Halosperm kit was from Halotech DNA (Madrid, Spain). MitoSOX Red and LIVE⁄DEAD Fixable Green Dead Cell Stain (LD-G) were from Thermo Fisher Scientific (Waltham, MA, USA). All the other reagents were from Merck Life Science, Milan, Italy.

### 2.2. Study Population and Semen Collection

Semen samples were collected consecutively among male partners of infertile couples (hereon indicated as subfertile patients) attending the Semen Cryopreservation and Andrology Laboratory of Careggi Hospital to undergo routine semen analysis from April 2023 to May 2024. Men with azoospermia or an insufficient sperm number for executing determination of oxidative stress (<0.5 million available) were excluded. In the recruited subjects, we detected sperm ROS production (n = 131) and sDF (n = 127). For control, we recruited 31 healthy donors who were selected among volunteers by administrating a structured questionnaire aimed at collecting information on any condition which might induce semen oxidative stress. A daily sedentary time higher than 8 h/day, occupational exposure to toxicants or high temperature, smoking habits, daily alcohol consumption, cryptorchidism and varicocele, occurrence of urogenital infections within 6 months, drug consumption and current disease were exclusion criteria. We also excluded, from the control group, men with leukocytospermia, semen viscosity and semen bacteria.

Written informed consent was obtained from participants. The study was approved by the ethical committee of AOU Careggi (protocol No. 15693/CAM_BIO).

### 2.3. Routine Semen Analysis

Semen sample collection and routine semen analysis were conducted according to the WHO guidelines [44]. Briefly, sperm concentration was assessed in formalin-diluted samples by a Neubauer-improved cell counting chamber; sperm motility was scored by distinguishing progressive, non-progressive and immotile spermatozoa in at least 200 cells; and Diff-Quick staining was used for assessing sperm morphology in at least 200 spermatozoa. Semen pH and semen volume were determined using a pH paper and weighting the sample, respectively. Detection of leukocytes was conducted when round cells exceeded 1 million/mL using pre-stained slides Testsimplets^®^ (AB Analitica, Padua, Italy) then evaluated at microscope with a 100x objective. We took a concentration of leukocytes ≥1 × 10^6^/mL as threshold for leukocytospermia. Agglutinates and aggregates were determined by checking the presence of motile spermatozoa sticking, respectively, to each other and to cells or debris or immotile spermatozoa. Viscosity was assessed by aspirating semen with a pipette, allowing it to drop by gravity and verifying whether discrete drops or any thread were formed. The presence of bacteria was qualitatively evaluated by microscopic observation. The Semen Cryopreservation and Andrology Laboratory of Careggi Hospital participates in external quality control programs: United Kingdom National External Quality Assessment Service (NEQAS) and External Quality Assessment of Tuscany.

### 2.4. Determination of Semen Oxidative Stress

**Figure 1:**
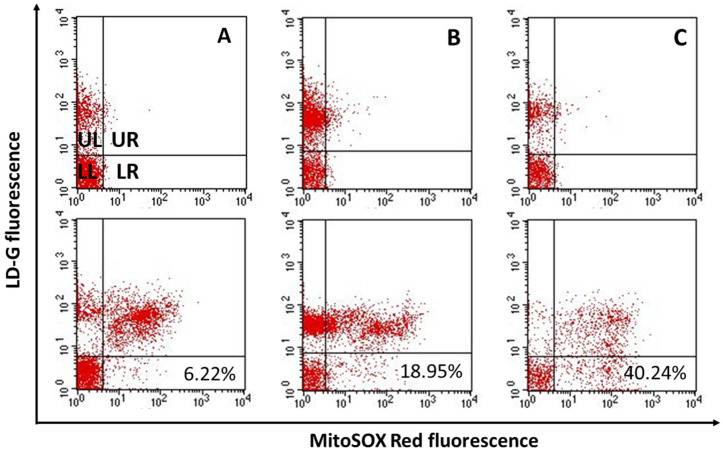
Sperm ROS production in semen samples. Representative MitoSOX Red/LD-G dot plots of a healthy donor (**A**), a subfertile patient (**B**) and a subfertile patients with semen bacteria (**C**). The percentage of oxidative stress is also reported for each example. Quadrant setting of each dot plot was established on the corresponding negative control (first row). LL, lower left quadrant; LR, lower right quadrant; UL, upper left quadrant; UR, upper right quadrant.

Oxidative stress was determined by double staining with MitoSOX Red and LD-G then detected by flow cytometry [40]. Briefly, semen samples (0.5–3 million of spermatozoa) were washed twice and then incubated in 500 µL PBS containing LD-G (1:10,000 dilution, 1 h at RT in the dark). After two washes with 200 μL of PBS, samples were split into two 100 µL aliquots which were incubated for 15 min at RT after adding (test sample) or not (negative control) 2 µM MitoSOX Red. After two further washes with PBS, samples were resuspended in 400 µL of PBS for acquisition with a flow cytometer (FACScan, BD Biosciences, San Jose, CA, USA) equipped with a 15-mW argon-ion laser for excitation. After proper compensation of the spillover of LD-G into MitoSOX Red or propidium iodide (PI, see below) channel and of MitoSOX Red into LD-G channel, LD-G was revealed by an FL-1 detector (515–555 nm wavelength band), whereas MitoSOX Red and PI were detected by an FL-2 detector (563–607 nm wavelength band). For each sample, 5000 LD-G negative events (i.e., viable spermatozoa) were recorded within a flame-shaped region (FR) drawn in the FSC/SSC dot plot. FR excludes debris and all non-sperm cells and contains spermatozoa and apoptotic bodies [45]. Apoptotic bodies are stained by LD-G and, thus, do not interfere with analysis of viable spermatozoa (LD-G negative) [40]. For data analysis, we established quadrants including about 1% of events in the Low Right quadrant of the MitoSOX Red/LD-G dot plot of negative control (Figure 1, upper panels). Hence, such quadrants were copied in the dot plot of the corresponding test sample (Figure 1, lower panels). Finally, we calculated oxidative stress as the percentage of viable spermatozoa with MitoSOX Red staining on total viable spermatozoa (lower right quadrant/lower right and lower left quadrants in the MitoSOX Red/LD-G dot plot, Figure 1). We also calculated the percentage of viable spermatozoa with MitoSOX Red staining on total (viable and non-viable) spermatozoa (hereon indicated as total oxidative stress, tOS). To determine total spermatozoa, after the first acquisition, we treated the negative control with digitonin (200 mg/mL) and PI (30 mg/mL) and, then, acquired it again by flow cytometer. Since PI stains spermatozoa but not apoptotic bodies, only the former are shifted towards high values of red fluorescence, allowing their exact identification (for other details, see [43]).

### 2.5. Determination of sDF

SDF was determined with SCD (Sperm Chromatin Dispersion) test using Halosperm kit, following manufacturer’s instructions with some modifications. Briefly, 50,000 spermatozoa were resuspended in 1% low melting point agarose, layered on pre-coated agarose slides and covered with coverslips. Hence, slides were kept at 4 °C for few minutes and then treated with the acid denaturation solution and the lysing solution, both provided by the kit. Then, we dehydrated samples with 70% and then 100% Ethanol and stained them with eosin and then thiazine (15 min at RT for each stain). After drying, sDF was determined by scoring spermatozoa without or with small halo in at least 200 spermatozoa/slide [46].

### 2.6. Statistical Analyses

For data analysis, we used Statistical Package for the Social Sciences for Windows (SPSS 29, Inc., Chicago, IL, USA). To check the normal distribution of the tested variables, we used a Kolmogorov–Smirnov test. Most variables exhibited a non-normal distribution; hence, data were expressed as median [interquartile range, IQR]. Statistical differences in age, abstinence, semen parameters, oxidative stress and sDF between subfertile patients and healthy donors were assessed by the Mann–Whitney U test. The same test was used to compare the values of oxidative stress, tOS and sDF between patients with and without leukocytospermia, viscosity, presence of agglutinates/aggregates and bacteria. Correlation analyses were performed calculating the Spearman’s coefficient and applying the Holm method for adjusting the *p* value for multiple comparisons. These last analyses were conducted using R software 4.4.0 with the following libraries: “correlation”, “ggstatsplot”, and “PerformanceAnalytics”.

## 3. Results

**Figure 2:**
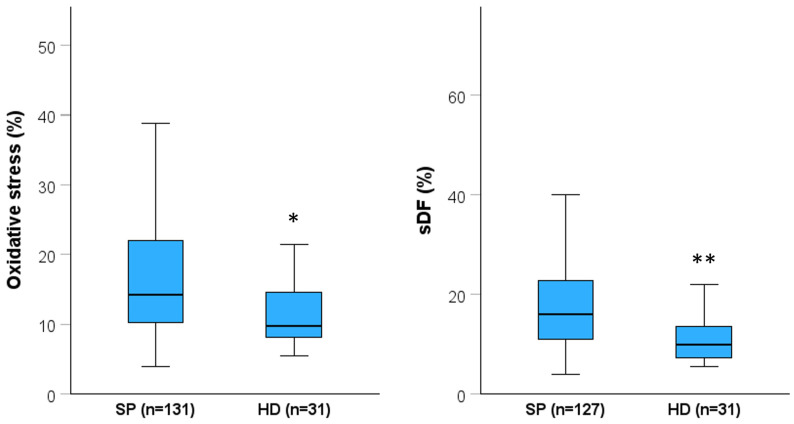
Oxidative stress and sDF as found in subfertile patients (SP) and healthy donors (HD). Box graphs report median [IQR] and minimum and maximum values (excluding outliers). * *p* (vs. HD) < 0.01; ** *p* (vs. HD) < 0.001; Mann-Whitney U-test.

In this study, we detected oxidative stress in native semen samples with a MitoSOX Red/LD-G double staining then revealed by flow cytometry. This method detects the viable sperm fraction with excessive ROS production and expresses oxidative stress as a percentage of the viable spermatozoa. We also calculated the percentage of viable spermatozoa with excessive ROS production on total (viable and non-viable) spermatozoa (tOS) (see Materials and Methods for further details). In Table 1, the values for age and conventional semen parameters of 131 consecutively recruited subfertile patients and of 31 healthy donors are reported. As shown, subfertile patients were older and with a worse motility (lower progressive motility and a higher percentage of immotile cells) than healthy donors. An increase in semen pH and abstinence length was also observed in subfertile patients, albeit remaining within a physiological range or the WHO guidelines indications, respectively. Finally, a trend towards a better sperm morphology was also observed in healthy donors. As shown in Figure 1 and Figure 2, subfertile patients also had higher values for oxidative stress (14.22 [10.21–22.08]%) and sDF (16.00 [11.00–23.50]%) than HD (oxidative stress: 9.75 [8.00–14.90]%, *p* < 0.01; sDF: 10.00 [7.00–14.00]%, *p* < 0.001), whereas similar values of tOS were found in the two groups (subfertile patients: 8.37 [6.50–13.28]% vs. healthy donors: 7.70 [6.00–10.61]%, *p* = 0.093).

Then, we studied the relationship of oxidative stress, tOS and sDF with age and conventional semen parameters by calculating the Spearman’s coefficient (Table 2). We found the expected significant correlations between sDF and progressive motility, percentage of immotile spermatozoa and sperm concentration [47,48]. In addition, sDF also correlated negatively with tOS. Conversely, oxidative stress showed no significant correlation with either age or conventional sperm parameters or sDF (Table 2).

**Figure 3:**
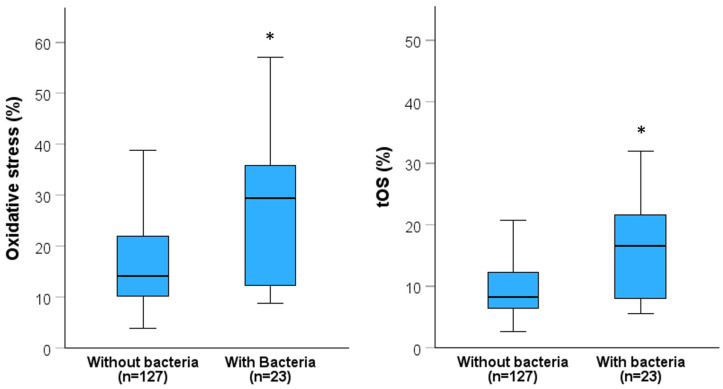
Oxidative stress and tOS as found in subfertile patients (SP) without and with semen bacteria. Box graphs report median [IQR] and minimum and maximum values (excluding outliers). *, *p* (vs. SP without bacteria) < 0.01; Mann-Whitney U-test.

Beside main semen and sperm parameters, routine semen analysis determines also leukocytospermia, viscosity, presence of agglutinates or aggregates and bacteriospermia. Since some of these characteristics have been associated to semen oxidative stress [49,50,51,52,53,54], we verified whether leukocytospermia (n = 7, 15.3%), viscosity (n = 35, 26.7%), presence of aggregates (n = 29, 22.1%) or agglutinates (n = 46, 35.1%) and bacteriospermia (n = 4, 3.1%) increased the values of oxidative stress, tOS and sDF. Results indicated that none of these signs changed the value of the tested variables (Table S1). An important exception was the presence of semen bacteria. Indeed, bacteriospermia did not affect sDF values but highly increased oxidative stress and tOS (Table S1), albeit without reaching the full statistical significance (*p* = 0.071 and *p* = 0.053, respectively), possibly because of the low number of subjects in the group with semen bacteria. Hence, to confirm the effect of bacteriospermia, we recruited an additional 19 subfertile patients with semen bacteria, reaching a total of 23 subjects (Table S2). Then, we compared the values of oxidative stress and tOS in patients with (n = 23) and without (n = 127) bacteriospermia. The results are reported in Figure 3, confirming that the presence of bacteria highly increased both oxidative stress (29.37 [11.18–36.00]% vs. 14.20 [1.12–22.00]%, *p* < 0.01) and tOS (16.62 [7.79–22.35] vs. 8.30 [6.48–12.46]%, *p* < 0.01). No difference was found in sDF values between the two groups (without: 16.00 [11.00–23.13]% vs. with: 16.50 [12.88–23.44]%, *p* = 0.246).

**Figure 4:**
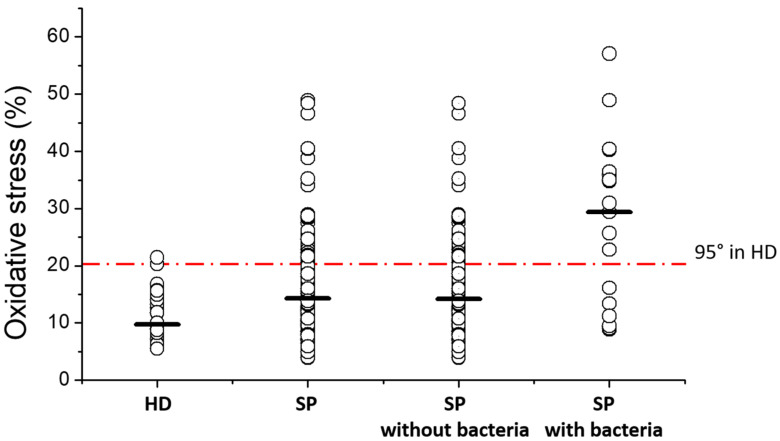
Frequency of subfertile patients (SP) with sperm ROS production above a threshold of 20.72%. The threshold was established as the 95th percentile in healthy donors and the number and percentage of SP exceeding the threshold were determined in the indicated groups. Thick bars represent the median values.

Finally, we established a threshold of oxidative stress, calculating the 95th percentile in healthy donors which resulted 20.72%. Among the consecutively recruited patients, we found that 29.00% (38 out of 131) showed values of oxidative stress above this threshold. This percentage decreased to 25.56% (35 out of 127) when we excluded men with semen bacteria. Conversely, when only patients with semen bacteria (n = 23) were considered, the percentage of men with a value of oxidative stress above the threshold increased to 60.87% (14 out 23) (Figure 4). When a similar threshold was calculated for tOS (17.54%), only 12.98% subfertile patients exceeded the threshold; the percentage decreased to 12.60% when patients with semen bacteria were excluded, and increased to 43.48% when only patients with bacteria were considered.

## 4. Discussion

In this study, we detected oxidative stress with a novel flow cytometric method in subfertile patients, finding higher levels of the parameter in these patients than in the control group of healthy donors. After establishing a threshold as the 95th percentile of oxidative stress in healthy donors, we found that 29% of subfertile patients had values exceeding this threshold and that the percentage highly increased when only patients with semen bacteria were considered. We also found that oxidative stress did not correlate with either age or conventional semen parameters or sDF amounts, suggesting that the novel method detects a parameter independent from routine semen analysis and even from sDF determination. Hence, the novel method appears to promise additional information on male fertility status with respect to the current used semen parameters.

The lack of correlation with routine semen parameters is in contrast to many previous studies reporting an increase of oxidative stress with poor semen quality. Several of these studies used luminol or lucigenin as probes emitting light upon oxidation [55,56] or malondialdehyde detection [57,58] for evaluating oxidative stress in native semen samples after washing away semen plasma. Hence, these studies provided average measures of oxidative stress affected not only by spermatozoa but also by the amount of non-sperm elements (cells and apoptotic bodies), which increases with worsening semen quality [59,60]. Although we cannot conclude that these elements could increase oxidative stress, these measures are heavily biased by different contents in such elements depending on semen quality. Conversely, our study used flow cytometry, providing individual measures coming only from the viable sperm fraction of native semen samples, guaranteeing a more accurate detection of sperm oxidative stress. On the other hand, the reported associations between semen quality and oxidative stress as assessed by evaluation of the oxidation-reduction potential (ORP) [61,62,63] are only apparent. Indeed, such associations are driven by ORP normalization to sperm concentration and, thus, by the internal correlations between sperm concentration itself and the other semen parameters [39,64]. This is true also for the reported correlations between ORP and sDF [17], as the amount of sperm DNA breakage well correlates with semen quality and, in particular, with sperm concentration (present study and [47,48]). The lack of correlation between oxidative stress and sDF found in our study is not surprising also because sDF was detected in native semen samples. Indeed, in these specimens, sDF mainly is due to non-viable spermatozoa and associates to abortive apoptosis and/or defects in chromatin maturation [41,65], whereas no correlation is detected with signs of oxidative attack [41,66]. Only when sDF is detected in viable spermatozoa a large concomitance with oxidative damage can be observed [41,67]. Overall, our data indicate, for the first time, that excessive sperm ROS production, at least when measured by our method, may not be associated to idiopathic male infertility. Conversely, the independence of this parameter from any semen markers until now used in the clinical practice appears to promise additional information for, in particular, men with unexplained male infertility. The costs and need of experts of flow cytometry may, however, limit the practical utility of the method used in this study.

Beside assessing the main sperm parameters, routine semen analysis detects several semen traits that have been associated to oxidative stress, like viscosity [49,50,51], leukocytospermia [52,53] and bacteriospermia [54]. We did not find any increase of sperm ROS production in semen showing viscosity, a result that is in agreement with Layali et al., 2015 [68] but in contrast with other studies reporting a positive correlation between viscosity and levels of MDA and protein carbonyl [50] or a decrease of semen viscosity after treatment with the antioxidant N-acetylcysteine [69]. These discrepancies could be attributed to different methods to detect oxidative stress and semen viscosity. In particular, it has been reported that measuring viscosity with a capillary tube viscosimeter identified as hyperviscous a subgroup of samples classified as normoviscous by the WHO guidelines [50]. This finding shows that the viscosimeter is more accurate than the procedure indicated by the WHO manual [50,70] and used in this study [44].

In this study, we also failed to reveal an increase of sperm ROS production in samples where leukocyte concentrations were higher than 1 million/mL. Leukocytes are believed a major source of semen ROS, responsible for impairing sperm functions and structures. In particular, ROS can trigger sperm lipid peroxidation [71], which, in turn, stimulates mitochondrial superoxide generation [72]. However, the biological meaning of semen leukocyte in semen is a subject of some debate, with several studies failing to reveal its impact on semen quality [73,74,75]. In addition, there are conflicting opinions about the cut-off value of 1 million/mL as indicative of infections or inflammatory processes when exceeded [53]. These controversies are likely explained by the fact that several cell subsets of leukocytes are present in semen and only the activated ones can exert detrimental effects on spermatozoa [76]. In this scenario, it is not surprising that we did not find an increase of sperm ROS production in samples with leukocytes. However, we cannot rule out that recruiting a higher number of subjects with leukocytospermia than that included in this study (n = 7) might unveil the impact of this semen trait on oxidative stress.

Contrary to the results obtained for viscosity and leukocytospermia, we found that the presence of bacteria in semen highly increased sperm ROS production, a finding confirming previous studies on humans [53] and animals [77,78,79]. As explained in a recent review [54], bacteria can induce semen ROS not only by stimulating leukocytes, mainly polymorphonuclear neutrophilic granulocytes [80,81], but also through damaged spermatozoa [82] and bacterial metabolites/products [83,84]. In our study, we did not find a large concomitance between leukocytospermia and semen bacteria (Table S2), suggesting that a direct action on spermatozoa could be responsible of the observed increase in sperm ROS production in subjects with semen bacteria.

As mentioned, in this study, we found that in the male population undergoing routine semen analysis, 29% showed levels of oxidative stress higher than a threshold established in healthy donors. When only men without semen bacteria were considered, the percentage decreases to 25.56%. In these men, the increase of sperm ROS production might be due to lifestyle factors or exposure to pollution or endogenous conditions. Indeed, studies on animal models have extensively shown that obesity [12,13], tobacco [19,20] and alcohol abuse [21,22,23], prolonged sitting [24,25], recreational drugs [14,26], poor nutritional diet [15,16], varicocele [27], psychological stress [17,18], exposure to excessive heat [8,9], endocrine disrupters [10,11] and ionizing/nonionizing radiation [6,7] are all conditions which can increase semen oxidative stress. In addition, these findings have also been confirmed in humans, as recently reviewed [85,86]. In a fraction of this 25.56%, it is also possible that the observed high levels of oxidative stress were due to a presence of semen bacteria that we could not detect because of the qualitative evaluation of bacteriospermia (see below for further discussion on this point).

In this study, we also measured viable spermatozoa with excessive ROS production as percentage of total (viable and non-viable) spermatozoa (tOS). This parameter, however, failed to reveal a difference in sperm ROS production between subfertile patients and the control group. In addition, a lower number of patients exceeded the threshold established on this parameter with respect to the percentage calculated on only viable spermatozoa. These results might be caused by the fact that tOS values are decreased by non-viable spermatozoa, which likely are more present in subfertile patients than in healthy donors. Conversely, oxidative stress expressed as percentage of only viable spermatozoa is totally independent from sperm viability. The decrease in tOS with the amount of non-viable spermatozoa also explains the negative correlation of the parameter with sDF (Table 2), which, when detected in native semen samples, is near completely due to dead spermatozoa [41,65,87]. Overall, the current results confirm a previous study [43] indicating a lower sensitivity of tOS for the detection of sperm oxidative stress.

One major limitation of this study is that bacteriospermia were assessed qualitatively by microscopic evaluation, as routine semen analysis conducted according to the WHO guidelines does not include quantitative detection of semen bacteria [44]. Since we found that semen bacteria did not necessarily increase sperm ROS production (Figure 4), further studies are necessary for assessing (i) whether the increase of oxidative stress occurs only when bacteria exceed a certain threshold (for instance, 10^3^ colony-forming units/mL) [53,88] and (ii) which bacterial species are able to induce excessive sperm ROS production. Another limitation of the study is that we could not collect any information on female factors of couple infertility; thus, it is possible that a certain percentage of fertile men could be present in the recruited male population. If so, given the importance attributed to sperm oxidative stress for male infertility, the percentage of subfertile patients exceeding the threshold of 20.72% might be higher than that found in the study. Finally, we do not have any information on the presence of environmental, lifestyle and endogenous factors in the recruited patients. Hence, we cannot definitely conclude that one or more of these factors are responsible for the increase in sperm oxidative stress observed in a relevant fraction of subfertile men.

In conclusion, we showed here that 29% of men undergoing routine semen analysis exhibit high values of sperm ROS production, meaning that these values exceeded a threshold of 20.72% established in healthy donors. The amounts of oxidative stress did not correlate to either age or conventional semen parameters or sDF, suggesting that sperm ROS production detected by the flow cytometric method used in this study is independent from semen parameters until now used in the clinical practice. The presence of bacteria in semen highly increased sperm ROS production at variance with viscosity and leukocytospermia. Factors related to lifestyle, exposure to pollutants and endogenous conditions might be responsible for excessive sperm ROS production in men without semen bacteria.

## Figures and Tables

**Table 1:** Age, abstinence and main semen parameters in subfertile patients and healthy donors. SP, subfertile patients; HD, healthy donors. Data are median [IQR]. Mann-Whitney U-test.

Parameter	SP n = 131	HD n = 31	*p*-Values
Age (y)	35.00 [29.00–42.00]	29.00 [25.00–32.00]	<0.001
Abstinence (d)	4.00 [3.00–5.00]	3.00 [2.00–5.00]	00.012
Volume (mL)	3.80 [2.80–4.80]	3.60 [2.30–4.60]	0.375
pH	7.60 [7.60–7.80]	7.40 [7.20–7.60]	<0.001
Concentration (10^6^/mL)	57.00 [23.80–96.00]	83.00 [40.00–100.00]	0.112
Number (10^6^/ejaculate)	205.84 [87.00–338.00]	228.80 [138.56–393.30]	0.194
Progressive Motility (%)	49.00 [36.00–60.00]	57.00 [51.00–65.00]	0.002
Immotile (%)	41.00 [32.00–54.00]	32.00 [25.00–40.00]	<0.001
Normal Morphology (%)	4.00 [2.00–6.00]	4.00 [3.00–7.00]	0.063

**Table 2:** Spearman’s correlation coefficients of the associations between oxidative stress or tOS or sDF and age, abstinence and semen parameters.

Rho Coefficient (95%CI)	Oxidative Stress	*p*-Values	tOS	*p*-Values	sDF	*p*-Values
Age	0.07 (−0.11–0.24)	0.450	0.11 (−0.07–0.28)	0.225	0.06 (−0.12–0.24)	0.480
Abstinence	0.08 (−0.10–0.25)	0.450	0.11 (−0.07–0.28)	0.225	0.09 (−0.09–0.27)	0.297
Volume	0.15 (−0.03–0.31)	0.095	0.06 (−0.11–0.24)	0.466	0.18 (0.00–0.35)	0.040
pH	−0.02 (−0.19–0.16)	0.861	−0.06 (−0.24–0.12)	0.485	−0.02 (−0.20–0.16)	0.808
Concentration	−0.14 (−0.31–0.04)	0.116	0.01 (−0.16–0.19)	0.867	−0.18 (−0.35–0.00)	0.038
Number	−0.06 (−0.24–0.11)	0.461	0.05 (−0.13–0.22)	0.571	−0.11 (−0.29–0.07)	0.210
Progressive Motility	−0.11 (−0.29–0.06)	0.191	0.15 (−0.03–0.32)	0.087	−0.37 (−0.51–0.20)	<0.001
Immotile	0.13 (−0.05–0.30)	0.141	−0.12 (−0.29–0.06)	0.171	0.32 (0.15–0.47)	<0.001
Normal Morphology	0.07 (−0.11–0.24)	0.460	0.17 (−0.01–0.34)	0.051	−0.07 (−0.24–0.12)	0.465
Oxidative Stress	/		0.82 (0.75–0.87)	0.000	0.07 (−0.11–0.24)	0.461
tOS	0.82 (0.75–0.87)	<0.001	/		−0.21 (−0.37–0.03)	0.018
sDF	0.07 (−0.11–0.24)	0.461	−0.21 (−0.37–0.03)	0.018	/	

*p* value adjustment for multiple comparisons (Holm method).

## Data Availability

The datasets generated during and/or analysed during the current study are available from the corresponding author on reasonable request.

## Supplementary Materials

The following supporting information can be downloaded at: https://www.mdpi.com/article/10.3390/antiox13091123/s1, Table S1: Impact of leukocytospermia, agglutinates, aggregates, viscosity and bacteriospermia on oxidative stress, tOS and sDF in the 131 consecutively recruited subfertile patients; Table S2: Age, abstinence and semen parameters in subfertile patients with semen bacteria.
