# Supplementary material for: Reliable Detection of Excessive Sperm Ros Production in Subfertile Patients: How Many Men with Oxidative Stress?
Source: Antioxidants (Basel). 2024 Sep 18;13(9):1123. doi: 10.3390/antiox13091123 (PMC11429313; doi:10.3390/antiox13091123)
Supplement: Supplementary file 1 [file antioxidants-13-01123-s001.zip › Table S1.pdf]

Supplementary Table S1. Impact of leukocytospermia, agglutinates, aggregates, viscosity and bacteriospermia on oxidative stress, tOS and sDF in the 131 consecutively recruited subfertile patients.

	Oxidative stress			tOS			sDF		
	with	without	p-values	with	without	p-values	with	without	p-values
**leukocytospermia**	16.00 [9.10-28.34] n=7	14.21 [10.25-21.97] n=124	.786	7.72 [6.20-17.51] n=7	8.38 [6.50-12.88] n=124	.992	11.00 [8.00-18.00] n=7	16.00 [11.00-23.87] n=120	.188
**agglutinates**	14.03 [9.38-21.40] n=46	14.68 [10.62-23.05] n=85	.421	7.81 [6.23-13.95] n=46	8.39 [6.72-13.15] n=85	.592	16.00 [11.00-21.00] n=46	16.00 [11.00-24.50] n=81	.960
**aggregates**	13.87 [9.58-22.04] n=29	14.54 [10.50-22.35] n=102	.508	7.61 [6.09-13.89] n=29	8.64 [6.50-13.42] n=102	.616	15.00 [10.50-20.00] n=29	16.00 [11.00-21.12] n=98	.350
**viscosity**	15.50 [11.00-20.25] n=35	14.09 [9.95-20.37] n=96	.099	8.69 [6.50-17.21] n=35	8.29 [6.42-12.19] n=96	.377	8.53 [6.50-17.28] n=34	16.00 [11.00-24.25] n=93	.641
**bacteriospermia**	31.60 [14.08-46.77] n=4	14.20 [10.12-21.90] n=127	.071	16.38 [10.31-21.50] n=4	8.20 [6.41-12.39] n=127	.053	17.00 [16.00-] n=3	16.00 [11.00-23.12] n=124	.270

tOS, oxidative stress as percentage in the total spermatozoa; sDF, sperm DNA fragmentation. Data are median[IQR]. Mann-Whitney U-test.
