# Supplementary material for: Reliable Detection of Excessive Sperm Ros Production in Subfertile Patients: How Many Men with Oxidative Stress?
Source: Antioxidants (Basel). 2024 Sep 18;13(9):1123. doi: 10.3390/antiox13091123 (PMC11429313; doi:10.3390/antiox13091123)
Supplement: Supplementary file 1 [file antioxidants-13-01123-s001.zip › Table S2.pdf]

Supplementary Table S2. Age, abstinence and semen parameters in subfertile patients with semen bacteria.

Parameter	n=23
**Age (y)**	33.00 [2.00-38.00]
**Abstinence (d)**	4.00 [3.00-5.00]
**Volume (ml)**	3.90 [2.42-4.80]
**pH**	7.60 [7.40-7.80]
**Concentration (106/ml)**	37.50 [16.00-66.00]
**Number (106/ejaculate)**	118.65 [47.50-184.90]
**Progressive Motility (%)**	47.00 [38.00-55.00]
**Immotile (%)**	42.00 [40.00-53.00]
**Normal Morphology (%)**	4.00 [2.00-7.00]
**leukocytospermia**	1, 4.3%
**viscosity**	9, 39.1%
**agglutinates**	5, 21.7%
**aggregates**	4, 17.4%
**bacteriospermia**	23, 100 %

Data are median[IQR]. For categorical variables, both number of patients and percentage are presented.
